# Difference of Sagittal Alignment between Adolescents with Symptomatic Lumbar Isthmic Spondylolisthesis and the General Population

**DOI:** 10.1038/s41598-018-29260-6

**Published:** 2018-07-19

**Authors:** Jian Zhao, Yongqiang Xiao, Xiao Zhai, Ziqiang Chen, Ming Li

**Affiliations:** 1 Department of Orthopedics, Changhai Hospital, Second Military Medical University, Shanghai, 200438 People’s Republic of China; 2 Department of Burn Surgery, Changhai Hospital, Second Military Medical University, Shanghai, 200438 People’s Republic of China

## Abstract

This case-control study aimed to investigate differences in the sagittal spinal parameters between the **symptomatic** spondylolisthesis patients and the general population. Twenty-nine adolescent patients with **symptomatic** lumbar isthmic spondylolisthesis were included. For each patient, two age-matched, gender-matched and BMI-matched controls were enrolled. Comparison analyses detected higher values in the case group for the following parameters: CL (−22.06 ± 7.552° versus −20.36 ± 7.016°, P < 0.001), T1 Slope (19.84 ± 8.708° versus 13.99 ± 6.537°, P = 0.001), PT (21.54 ± 9.082° versus 8.87 ± 7.863°, P < 0.001), PI (64.45 ± 13.957° versus 43.60 ± 9.669°, P < 0.001), SS (42.90 ± 9.183° versus 34.73 ± 8.265°, P < 0.001), LL (−50.82 ± 21.596° versus −43.78 ± 10.356°, P = 0.042), SVA (16.99 ± 14.625 mm versus 0.32 ± 31.824 mm, P = 0.009), L5 Slope (33.95 ± 13.567° versus 19.03 ± 6.809°, P < 0.001), and L5I (8.90 ± 6.556° versus 1.29 ± 6.726°, P < 0.001). Conversely, TS-CL (6.56 ± 6.716° versus 11.04 ± 7.085°, P = 0.006), cSVA (11.31 ± 6.867 mm versus 17.92 ± 11.832 mm, P = 0.007), and TLK (−2.66 ± 10.101° versus 2.71 ± 7.708°, P = 0.007) were smaller in the case group. Slippage percentage was most correlated with PI (r = 0.530, P = 0.003), followed by PT (r = 0.465, P = 0.011) and L5I (r = 0.433, P = 0.019). Results of binary logistic regression showed that the main risk factor of isthmic spondylolisthesis was PI (OR = 1.145, 95%CI = 1.083–1.210, P < 0.001). Further subgroup analysis also showed that PI was the main risk factor of isthmic spondylolisthesis in the female adolescents (OR = 1.237, 95%CI = 1.086–1.493, P = 0.003) and in the male adolescents (OR = 1.523, 95%CI = 1.093–2.123, P = 0.013). PI was the main risk factor for adolescent **symptomatic** isthmic spondylolisthesis in the Chinese Han adolescents. The greater PI indicated the higher the progressive risk of spondylolisthesis. In these isthmic spondylolisthesis adolescents, the body always inclined forward and lumbar and cervical lordosis increased.

## Introduction

Adolescents suffering from lumbar isthmic spondylolisthesis or spondylolysis always complain of low back pain^1^. Isthmic spondylolysis is defined as a unilateral or bilateral defect in the pars interarticularis, which frequently occurs at L4 or L5 vertebrae^2^. Based on different populations, the incidence of spondylolysis varied from 4.4% to 39.7%^3,4^. Adolescent athletes were more susceptible to isthmic spondylolysis^5^. Several factors were associated with the susceptibility to spondylolysis, including ethnic heterogeneity^6^, genetic background^7^, occupation^5^, and sagittal balance^8^. Spondylolisthesis is defined as the forward slippage of vertebrae with respect to the underlying vertebrae, which frequently occurs in patients with bilateral pars defects. This abnormality usually occurred after walking age, and the adaptation both in the sagittal balance of the spine and lumbar-pelvic-femoral complex was associated with spondylolisthesis. Furthermore, it was assumed that the acquisition of bipedalism coupled with vertical stance was the prerequisite to spondylolisthesis^8^.

In adults, several studies have investigated the sagittal alignment in different populations^9^. Sagittal spine alignment was associated with Health-related quality of life (HRQoL)^10^, especially in elderly individuals^11,12^. Based on 492 consecutive Caucasian patients, aged on average 51.9 ± 16.8 years, Schwab *et al*.^12^ observed that threshold values for severe disability (Oswestry Disability Index, ODI > 40) were comprised of (pelvic tilt, PT ≥ 22°), (sagittal vertical alignment, SVA ≥ 47 mm), and (pelvic incidence minus lumbar lordosis, PI-LL ≥ 11°). It is well-known that sagittal spinal alignment changes with age, and pelvic-spine parameters vary in different populations. For example, a relatively smaller pelvic incidence (PI) was observed in the Chinese Han population than in Caucasian populations^13,14^.

In adolescents where no aforementioned clinical evidence was observed, previous radiographic studies proposed that the average values of PT, PI, sacral slope(SS), and other sagittal parameters should be slightly smaller when compared with normal adults^15,16^. Insufficient information was reported regarding the normal sagittal alignment range in adolescents, especially concerning the association of abnormal sagittal alignment with lumbar diseases such as lumbar isthmic spondylolisthesis. Therefore, this study aimed to detect the difference of sagittal alignment between adolescent symptomatic lumbar isthmic spondylolisthesis patients and general adolescents in the Chinese Han population.

## Result

### General information

There were 13 male and 16 female adolescents in the case group. The control group comprised of 26 males and 32 females. The averaged slip percentage was 36.97 ± 8.95%. No difference was observed in terms of age (P = 0.780), height (P = 0.332), weight (P = 0.256), BMI (P = 0.798), and gender distribution (P = 0.589). The average age in the case and control groups was 14.03 ± 1.50 years and 14.14 ± 1.68 years, respectively. The average height was 155.10 ± 11.95 cm and 157.57 ± 10.69 cm, the average weight was 53.38 ± 7.55 kg and 55.33 ± 7.36 kg, and the average BMI was 22.15 ± 1.52 kg/m^2^ and 22.34 ± 1.45 kg/m^2^, respectively. Table 1 demonstrates the details on demographic characteristics.

**Table 1:** Demonstrated the details of demographic characteristics.

Variables	Case group (n = 29)	Control group (n = 58)	Value of P
Age (years)	14.03 ± 1.50	14.14 ± 1.68	0.780
Gender (male/female)	13/16	26/32	0.789
Height (cm)	155.10 ± 11.95	157.57 ± 10.69	0.332
Weight (kg)	53.38 ± 7.55	55.33 ± 7.36	0.256
Body Mass Index (kg/m^2^)	22.15 ± 1.52	22.34 ± 1.45	0.798
Diagnosis of isthmic defect (Oblique X-ray Films/CT images)	6/23	—	—
Slippage Percentage (%)	36.97 ± 8.95	—	—

### Univariate Analysis

Comparison analyses detected higher values in the case group for the following parameters cervical lordosis (CL: −22.06 ± 7.552° versus −20.36 ± 7.016°, P < 0.001), T1 Slope (19.84 ± 8.708° versus 13.99 ± 6.537°, P = 0.001), PT (21.54 ± 9.082° versus 8.87 ± 7.863°, P < 0.001), PI (64.45 ± 13.957° versus 43.60 ± 9.669°, P < 0.001), SS (42.90 ± 9.183° versus 34.73 ± 8.265°, P < 0.001), LL (−50.82 ± 21.596° versus −43.78 ± 10.356°, P = 0.042), SVA (16.99 ± 14.625 mm versus 0.32 ± 31.824 mm, P = 0.009), L5 Slope (33.95 ± 13.567° versus 19.03 ± 6.809°, P < 0.001), and L5 Incidence (L5I: 8.90 ± 6.556° versus 1.29 ± 6.726°, P < 0.001). Conversely, T1 Slope minus Cervical Lordosis (TS-CL: 6.56 ± 6.716° versus 11.04 ± 7.085°, P = 0.006), C2-C7 plumbline (cSVA: 11.31 ± 6.867 mm versus 17.92 ± 11.832 mm, P = 0.007), and thoracolumbar kyphosis (TLK: −2.66 ± 10.101° versus 2.71 ± 7.708°, P = 0.007) were smaller in the case group. There was no difference in C1-C2 cervical lordosis (C1-C2, P = 0.301), or thoracic kyphosis (TK, P = 0.844). Table 2 demonstrates the details on the comparison analyses.

**Table 2:** Demonstrated the details of the comparison analyses.

Parameters	Group	The whole group (n = 87)			The male group (n = 39)			The female group (n = 48)		
		N	Mean ± SD	Value of P	N	Mean ± SD	Value of P	N	Mean ± SD	Value of P
C1C2 (°)	Case	29	−22.06 ± 7.552	0.301	13	−22.27 ± 5.943	0.890	16	−21.89 ± 8.8391	0.240
	Control	58	−20.36 ± 7.016		26	−21.97 ± 6.517		32	−19.05 ± 7.2323	
C2-C7 (°)	Case	29	−13.28 ± 9.527	<0.001	13	−11.73 ± 8.608	0.010	16	−14.54 ± 10.314	<0.001
	Control	58	−2.78 ± 10.107		26	−2.17 ± 11.056			−3.28 ± 9.417	
T1 Slope (°)	Case	29	19.84 ± 8.708	0.001	13	16.31 ± 6.841	0.397	16	22.72 ± 9.189	<0.001
	Control	58	13.99 ± 6.537		26	14.40 ± 6.394		32	13.66 ± 6.734	
TS-CL (°)	Case	29	6.56 ± 6.716	0.006	13	4.55 ± 6.688	0.010	16	8.19 ± 6.486	0.247
	Control	58	11.04 ± 7.085		26	11.85 ± 8.359		32	10.38 ± 5.913	
cSVA (mm)	Case	29	11.31 ± 6.867	0.007	13	15.53 ± 7.437	0.216	16	7.89 ± 3.979	0.004
	Control	58	17.92 ± 11.832		26	20.65 ± 13.615		32	15.70 ± 9.830	
PT (°)	Case	29	21.54 ± 9.082	<0.001	13	27.70 ± 6.589	<0.001	16	16.54 ± 7.728	<0.001
	Control	58	8.87 ± 7.863		26	12.36 ± 7.874		32	6.03 ± 6.719	
PI (°)	Case	29	64.45 ± 13.957	<0.001	13	76.75 ± 7.925	<0.001	16	54.46 ± 8.706	<0.001
	Control	58	43.60 ± 9.669		26	51.32 ± 9.289		32	37.33 ± 3.425	
SS (°)	Case	29	42.90 ± 9.183	<0.001	13	49.06 ± 6.089	0.001	16	37.90 ± 8.258	0.002
	Control	58	34.73 ± 8.265		26	38.96 ± 8.984		32	31.29 ± 5.773	
LL (°)	Case	29	−50.82 ± 21.596	0.042	13	−49.38 ± 29.893	0.749	16	−51.99 ± 12.347	<0.001
	Control	58	−43.78 ± 10.356		26	−47.20 ± 12.454		32	−41.00 ± 7.371	
TLK (°)	Case	29	−2.66 ± 10.101	0.007	13	−10.48 ± 6.377	<0.001	16	3.69 ± 7.868	0.754
	Control	58	2.71 ± 7.708		26	2.40 ± 8.228		32	2.97 ± 7.382	
TK (°)	Case	29	21.52 ± 9.298	0.844	13	15.47 ± 6.342	0.038	16	26.44 ± 8.462	0.030
	Control	57	21.12 ± 8.894		26	21.69 ± 9.362		32	20.65 ± 8.467	
SVA (mm)	Case	29	16.99 ± 14.625	0.009	13	22.60 ± 17.288	0.215	16	12.43 ± 10.521	0.006
	Control	58	0.32 ± 31.824	.	26	8.95 ± 36.879		32	−6.69 ± 25.537	
L5 Slope (°)	Case	29	33.95 ± 13.567	<0.001	13	42.78 ± 7.153	<0.001	16	26.78 ± 13.421	0.001
	Control	58	19.03 ± 6.809		26	21.83 ± 6.977		32	16.75 ± 5.834	
L5 Incidence (°)	Case	29	−8.90 ± 6.556	<0.001	13	−13.32 ± 5.501	<0.001	16	−5.31 ± 5.033	0.004
	Control	58	−1.29 ± 6.726		26	−3.03 ± 7.032		32	0.13 ± 6.219	

For the male group, comparison analyses detected higher values in the case group for the following parameters including CL (−11.73 ± 8.608° versus −2.17 ± 11.056°, P = 0.010), PT (27.70 ± 6.589° versus 12.36 ± 7.874°, P =  < 0.001), PI (76.75 ± 7.925° versus 51.32 ± 9.289°, P < 0.001), SS (49.06 ± 6.089° versus 38.96 ± 8.984°, P = 0.001), L5 Slope (42.78 ± 7.153° versus 21.83 ± 6.977°, P < 0.001), and L5I (−13.32 ± 5.501° versus −3.03 ± 7.032°, P < 0.001). Conversely, TS-CL (4.55 ± 6.688° versus 11.85 ± 8.359°, P = 0.010) and TLK (−10.48 ± 6.377° versus 2.40 ± 8.228°, P < 0.001) were smaller in the case group. While there was no difference in C1-C2 (P = 0.890), T1 Slope (P = 0.397), cSVA (P = 0.216), LL (P = 0.749), and SVA (P = 0.215) (Table 2).

For the female group, comparison analyses detected higher values in the case group for the following parameters including CL (−14.54 ± 10.314° versus −3.28 ± 9.417°, P < 0.001), T1 Slope (22.72 ± 9.189° versus 13.66 ± 6.734°, P < 0.001), PT (16.54 ± 7.728°, versus 6.03 ± 6.719°, P < 0.001), PI (54.46 ± 8.706° versus 37.33 ± 3.425°, P = 0.002), LL (−51.99 ± 12.347° versus −41.00 ± 7.371°, P < 0.001), TK (26.44 ± 8.462° versus 20.65 ± 8.467°, P = 0.030), SVA (12.43 ± 10.521 mm versus −6.69 ± 25.537 mm, P = 0.006), L5 Slope (26.78 ± 13.421°, versus 16.75 ± 5.834°, P = 0.001), and L5I (−5.31 ± 5.033°, versus 0.13 ± 6.219°, P = 0.004). Conversely, cSVA (7.89 ± 3.979 mm versus 15.70 ± 9.830 mm, P = 0.004) was smaller in the case group. While there was no difference in C1-C2 (P = 0.240), and TLK (P = 0.754) (Table 2).

### Correlations between Slippage percentage and other sagittal spinal parameters

For the whole case and control groups, slippage percentage was most correlated with PI (r = 0.530, P = 0.003), followed by PT (r = 0.465, P = 0.011) and L5I (r = 0.433, P = 0.019). In the male group, slippage percentage was correlated with SVA (r = 0.568, P = 0.022). However, no significant correlation was observed in the female group (Table 3).

**Table 3:** Correlations between Slippage percentage and other sagittal spinal parameters.

Variable	The whole group (n = 87)		The male group (n = 39)		The female group (n = 48)	
	R	Value of P	r	Value of P	r	Value of P
C1C2	−0.271	0.155	−0.195	0.470	−0.451	0.122
C2C7	0.121	0.533	0.026	0.924	0.101	0.742
T1 Slope	−0.049	0.802	0.015	0.957	0.396	0.18
TS-CL	0.105	0.588	0.061	0.823	0.534	0.06
cSVA	0.418	0.024	0.282	0.290	0.106	0.73
PT	0.465	0.011	0.405	0.120	0.035	0.909
PI	0.530	0.003	0.220	0.413	0.162	0.597
SS	0.347	0.065	−0.148	0.585	0.173	0.572
LL	0.206	0.284	0.585	0.681	0.286	0.344
TLK	−0.379	0.042	0.145	0.593	−0.282	0.351
TK	−0.436	0.018	−0.114	0.674	−0.36	0.228
SVA	0.365	0.052	0.568	0.022	0.131	0.669
L5 Slope	0.341	0.070	0.014	0.960	0.268	0.376
L5 Incidence	0.433	0.019	−0.201	0.455	−0.103	0.737

### Multivariate Analysis

Results of binary logistic regression showed that the main risk factor of isthmic spondylolisthesis was PI (OR = 1.145, 95%CI = 1.083–1.210, P < 0.001) with the following parameters excluded (PT, SS, SVA, LL, L5I and L5 Slope, P > 0.05) (Table 4).

**Table 4:** Binary logistic regression analysis by forward stepwise (Conditional) for risk factors of adolescent lumbar isthmic spondylolisthesis.

Variable	B	Standard Error	Wald	df	P value	OR	95% CI
Constant	−7.781	1,525	26.031	1	<0.001		
PI	0.135	0.028	22.888	1	<0.001	1.145	1.083–1.210

Lumbar isthmic spondylolisthesis group was designated as 1, the control was as 0, in order to interpret the findings. The independent variables were PT, PI, SS, SVA, LL, L5I and L5 Slope.

In the male group, the main risk factor of isthmic spondylolisthesis was PI (OR = 1.237, 95%CI = 1.086–1.493, P = 0.003) with the following parameters excluded (PT, SS, TLK, TK, L5 Incidence and L5 Slope, P > 0.05) (Table 5).

**Table 5:** Binary logistic regression analysis by forward stepwise (Conditional) for risk factors of adolescent lumbar isthmic spondylolisthesis in the male.

Variable	B	Standard Error	Wald	df	P value	OR	95% CI
Constant	−16.287	5.525	8.690	1	0.003		
PI	0.242	0.081	8.872	1	0.003	1.273	1.086–1.493

Lumbar isthmic spondylolisthesis group was designated as 1, the control was as 0, in order to interpret the findings. The independent variables were PT, PI, SS, TLK, TK, L5 Incidence and L5 Slope.

In the female group, the main risk factor for isthmic spondylolisthesis was PI (OR = 1.523, 95%CI = 1.093–2.123, P = 0.013) with the following parameters excluded (PT, SS, LL, SVA, TK, L5 Incidence and L5 Slope, P > 0.05) (Table 6).

**Table 6:** Binary logistic regression analysis by forward stepwise (Conditional) for risk factors of adolescent lumbar isthmic spondylolisthesis in the female.

Variable	B	Standard Error	Wald	df	P value	OR	95% CI
Constant	−18.673	6.974	7.169	1	0.007		
PI	0.421	0.169	6.170	1	0.013	1.523	1.093–2.123

Lumbar isthmic spondylolisthesis group was designated as 1, the control was as 0, in order to interpret the findings. The independent variables were PT, PI, SS, LL, SVA, TK, L5 Incidence and L5 Slope.

## Discussion

To the best of our knowledge, no report has been focused on the sagittal spinal alignment in isthmic adolescent lumbar spondylolisthesis patients in the Chinese Han population. Comparison analyses detected higher values in our case group for the following parameters: CL (P < 0.001); T1 Slope (P = 0.001); PT (P < 0.001); PI (P < 0.001); SS (P < 0.001); LL (P = 0.042); SVA (P = 0.009); L5 Slope (P < 0.001); and L5I (P < 0.001). Correlation analysis detected that slippage percentage was most correlated with PI (r = 0.530, P = 0.003), followed by PT (r = 0.465, P = 0.011) and L5I (r = 0.433, P = 0.019). Binary logistic regression showed that the main risk factor of isthmic spondylolisthesis was PI (OR = 1.145, 95%CI = 1.083–1.210, P < 0.001).

It is commonly accepted that the sagittal spine misalignment plays a vital role in the mechanisms of several spine disorders. For example, it has been reported that young lumbar disc herniation patients demonstrated smaller LL, TK, and SS in the Chinese Han population^13^. In adults, Yin *et al*.^17^ reported that elevated PI and small sacral table angle (STA) played vital roles in lumbar spondylolysis in the Chinese Han population. In our study, the increased PI was also observed in adolescents with lumbar isthmic spondylolisthesis (64.45 ± 13.957° versus 43.60 ± 9.669°, P < 0.001). Previous studies reported that the PI in the Chinese Han population ranged from 40 to 50 degrees^18^, which was smaller than that found in Caucasian^15^ as well as in Korean populations^19^. Our study also detected a similar PI (43.60 ± 9.669°) in normal adolescents. In addition, this study also observed higher PT (21.54 ± 9.082° versus 8.87 ± 7.863°, P < 0.001) and SS (42.90 ± 9.183° versus 34.73 ± 8.265°, P < 0.001) in the case group, which was in accordance with the previous findings in adult spondylolisthesis patients^17^. In both the female and male group, the greater values of PI, PT, and SS also presented in lumbar isthmic spondylolisthesis patients.

Moreover, logistic regression analysis indicated that the main risk factor of isthmic spondylolisthesis was PI (OR = 1.145 95%CI = 1.083–1.210, P < 0.001), which was similar to findings in previous reports^15,17^. There was a significant correlation between PI and other sagittal parameters, including SS, PT and LL. PI increase was accompanied by an increase in both SS and PT, with a greater increase in SS than in PT. For those individuals of higher PI, larger LL always occurred to guarantee C7 plumb line behind the femoral head and to maintain the posture balance^20^. However, the larger LL and SS could exert relatively higher forward shear force on an isthmic of L5, which, coupled with the defect of pars interarticularis, would lead to lumbar spondylolisthesis. Moreover, the increased shear force also led to a larger L5 slope in the control group. Further subgroup analysis also detected that PI was the main risk factor of isthmic spondylolisthesis, both in female adolescents (OR = 1.237, 95%CI = 1.086–1.493, P = 0.003) and in male adolescents (OR = 1.523, 95%CI = 1.093–2.123, P = 0.013).

As for the LL, increased LL was observed in the case groups (−50.82° ± 21.596° versus −43.78° ± 10.356°, P = 0.042). Further subgroup analysis also detected higher LL in the female isthmic spondylolisthesis (−51.99 ± 12.347° versus −41.00 ± 7.371°, P < 0.001). Similarly, a greater degree of LL was also observed in isthmic spondylolisthesis patients (−55° ± 6°) than in degenerative spondylolisthesis patients (−43° ± 13°), as well as in the controls (−48° ± 12°) in Koreans (P = 0.004)^21^. LL is essential to maintain an upright posture in human being. Reports have shown that the lumbar lordosis angle increased until 14 or 16 years of age, and that the increased disc wedging angle resulted in the increase of LL^22^. Therefore, the relatively young average age might explain the smaller LL than previously reported LL values^17,18^. Similarly, the small TK and TLK values were also observed when compared with the corresponding values in adults.

Initially, Roussouly *et al*.^23^ introduced the L5I. It was inferred that L5I was significantly associated with isthmic spondylolisthesis. Based on 138 healthy volunteers, Zhu *et al*.^24^ reported mean values of L5I in adolescents (17.63 ± 8.65°) and adults (16.43 ± 7.64°). They also reported that there was a positive correlation between L5I and PI (r = 0.818), and established a linear formula to evaluate an ideal L5I based on PI (L5I = 0.725PI-12.757). Thus, the larger the PI, the larger also the L5I. Therefore, the greater L5I (8.90 ± 6.556° versus 1.29 ± 6.726°, P < 0.001) in the case group might result from the greater PI (64.45 ± 13.957° versus 43.60 ± 9.669°, P < 0.001). Additionally, the further subgroup analysis also demonstrated higher L5I values both in the male and female isthmic spondylolisthesis patients.

This study also compared the cervical sagittal alignment between the two groups. In the case group, we observed a greater T1 Slope (19.84 ± 8.708° *versus* 13.99 ± 6.537°, P = 0.001), and a greater CL (−22.06 ± 7.552° *versus* −20.36 ± 7.016°, P < 0.001). However, there was no difference in C1-C2 (P = 0.301). Smaller cSVA (11.31 ± 6.867 mm *versus* 17.92 ± 11.832 mm, P = 0.007) and smaller TS-CL (6.56 ± 6.716° versus 11.04 ± 7.085°, P = 0.006) were demonstrated in the case group. Recently, an increased number of studies was focused on cervical sagittal alignment. Hiyama *et al*.^25^ proposed that cervical sagittal alignment could be affected by thoracic deformity. Similarly, Hwang *et al*.^26^ demonstrated that there was a significant association between TK and cervical sagittal alignment. Moreover, another study suggested that the cervical sagittal alignment was correlated with the global sagittal spine alignment rather than regional thoracic kyphosis^27^. Given the larger SVA (16.99 ± 14.625 mm versus 0.32 ± 31.824 mm, P = 0.009) and T1 slope (19.84 ± 8.708° *versus* 13.99 ± 6.537°, P = 0.001) in the case group, we also inferred that the increased lordosis in cervical sagittal plane might compensate for the forward inclined trunk. So, the horizontal gaze can be guaranteed. In addition, the further subgroup analysis also demonstrated greater cervical lordosis in both the male and the female isthmic spondylolisthesis patients.

As for slippage percentage, correlation analysis detected that it was most correlated with PI (r = 0.530, P = 0.003), followed by PT (r = 0.465, P = 0.011) and L5I (r = 0.433, P = 0.019) (Table 3). Previously, Rajnics *et al*.^28^ reported a similar correlation coefficient (r = 0.660) between slip percentage and PI. Another study also reported that slip percentage was correlated with PI (r = 0.293, p = 0.023)^29^. Therefore, we inferred that progression of isthmic lumbar spondylolisthesis in adolescents was associated with a greater PI. Positive correlations were also detected in PT (r = 0.465, P = 0.011), L5I (r = 0.433, P = 0.019) and cSVA (r = 0.418, P = 0.024), which presumably results from the compensation for the forward inclined body to maintain sagittal balance. However, slippage percentage was only correlated with SVA (r = 0.568, P = 0.022) in the male population. In the female population, no significant correlation was observed. It should be noticed that the small sample size might have failed to reflect the correlation when subgroup analysis was performed based on gender difference.

Even though this study investigated the sagittal spinal alignment difference between the adolescent isthmic spondylolisthesis patients and the general population in China, several limitations should be taken into consideration. First, only 29 adolescent isthmic spondylolisthesis patients were included, thus the statistical power may be dwarfed. Second, it was understood that different ethnicities demonstrated different values of sagittal spinal parameters; therefore, further studies should be performed in additional ethnic populations. Third, spinal and pelvic parameters have been verified to be significantly associated with HRQoL, which may change with increasing age. This was not evaluated in our study, since no symptoms were observed in the controls.

## Conclusion

PI was the main risk factor for adolescent symptomatic isthmic spondylolisthesis in the Chinese Han adolescent population. The greater PI indicated the higher the progressive risk of spondylolisthesis. In isthmic spondylolisthesis adolescents, the body always inclined forward. With pelvic retroversion essential to maintaining sagittal balance, lumbar and cervical lordosis was always increased.

## Methods and Materials

**Figure 1:**
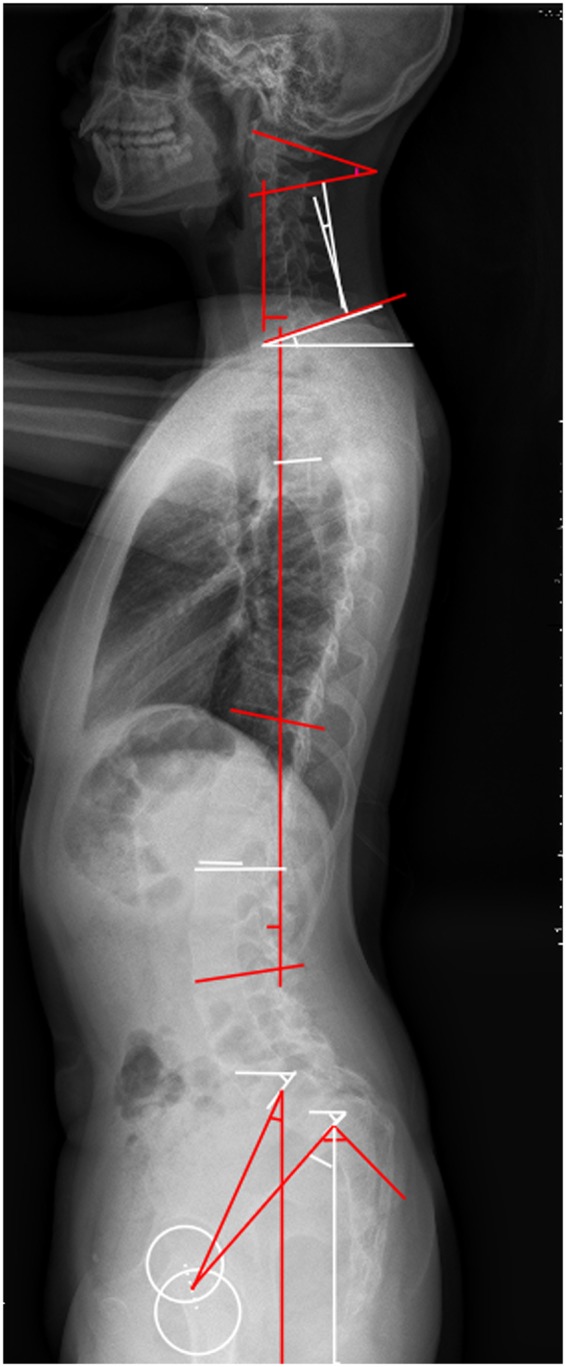
Demonstrated an adolescent lumbar isthmic spondylolisthesis patient.

From August 2009 to August 2017, a consecutive group of 29 adolescent patients with lumbar spondylolistheses was reviewed. All patients complained repeatedly of low back pain and were admitted to our department for surgical treatment. Oblique X-ray films or CT images were used to confirm the defects in the pars interarticularis (Fig. 1). For each patient, we selected 2 age-matched, gender-matched, Body Mass Index(BMI)-matched controls who attended the Outpatient Department for scoliosis screening, and were eventually excluded via full spine X-ray films. None of the controls had a history of spinal disorders or spine surgery, nor any history of low back pain and radiologic abnormalities. Moreover, all cases and controls were less than 18 years old. This study was approved by the Institutional Review Board of ChangHai hospital. This study was approved by the Institutional Review Board of ChangHai hospital. Given the fact that all participants were under the age of 18, informed consents were obtained from their legal guardians. We confirmed that all experiments were performed in accordance with relevant guidelines and regulations.

### Data collection

Demographic details on age, height, weight, BMI, and gender were collected. The radiographic films were measured independently by two researchers. The parameters measured were as follows:

1. Cervical sagittal alignment parameters: C1-C2 (C1-C2 cervical lordosis, the angle between C1 and the caudal endplate of C2), CL (C2-C7 cervical lordosis is the angle between the caudal endplate of C2 and the caudal endplate of C7), cSVA(cSVA is the horizontal offset from the plumbline dropped from C2 to the posterosuperior corner of C7), TS-CL (T1 Slope minus CL is the difference between T1 Slope and CL) (Fig. 2).

**Figure 2:**
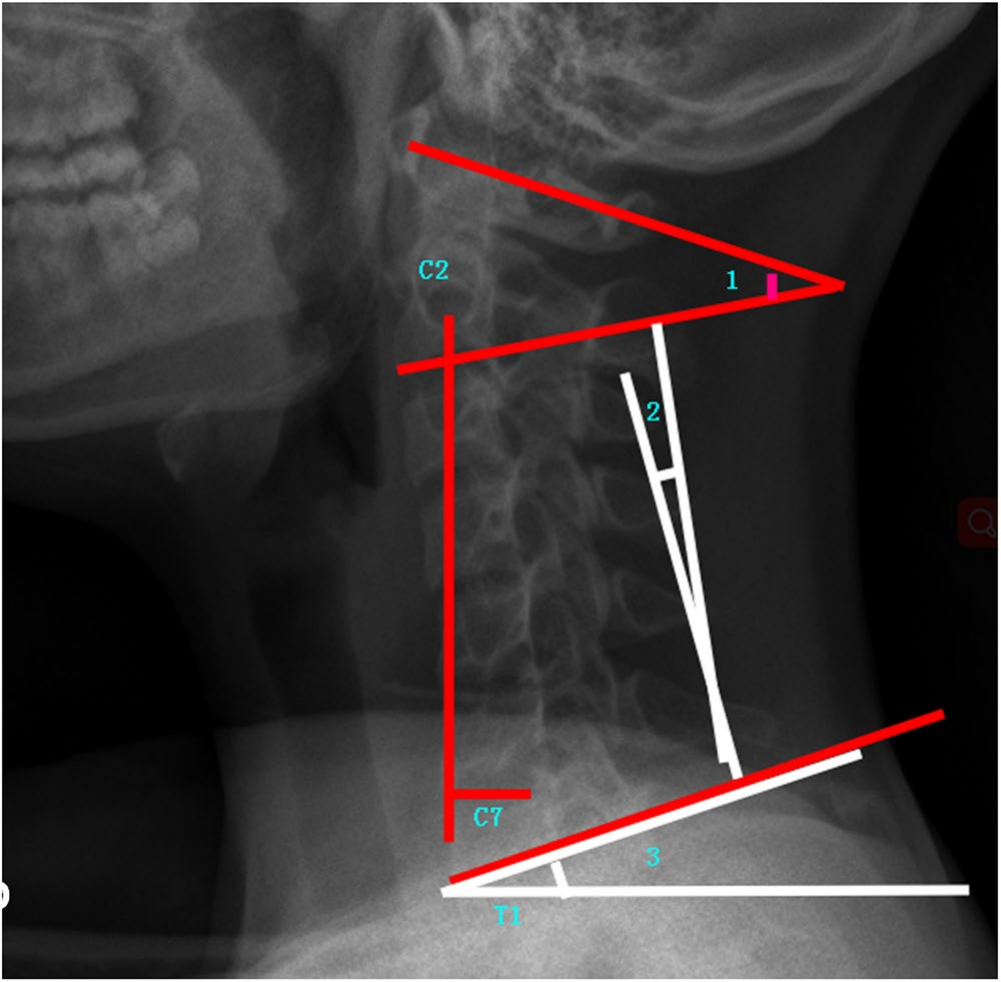
Demonstrated cervical sagittal alignment parameters (1:C1-C2, 2:C2-C7, and 3: T1 Slope).
2. Thoracic kyphosis and Lumbar lordosis parameters: TK (thoracic kyphosis)^30,31^, LL (lumbar lordosis)^30,31^, TLK (thoracolumbar kyphosis).
3. Sagittal lumbosacral parameters: SS, PT, PI, L5 Slope(the angle between a horizontal line and the superior end plate of L5), and L5I (L5 Incidence, the angle between the vertical line and the line connecting the center of the femoral heads axis to the center of the upper endplate of L5^23^) (Fig. 3).

**Figure 3:**
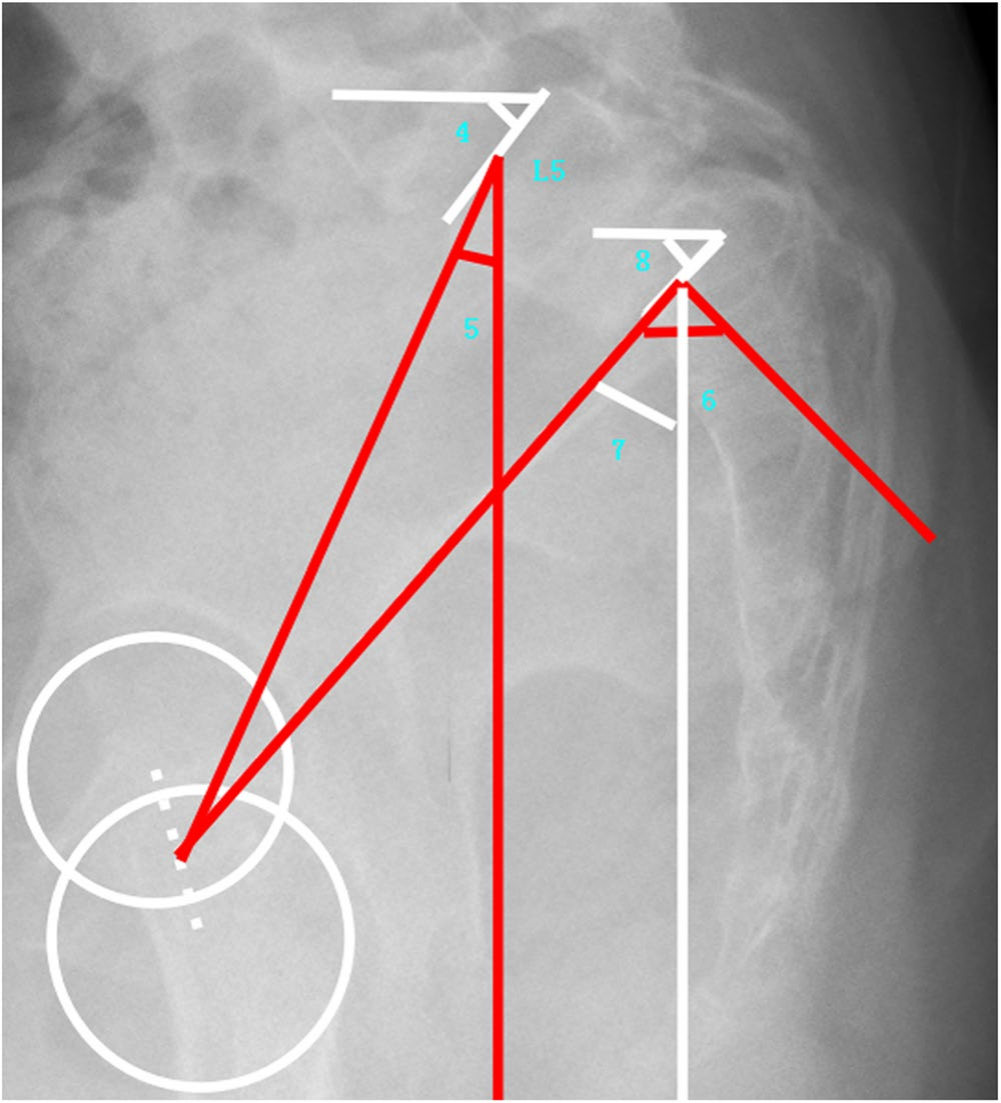
Demonstrated sagittal lumbosacral parameters (4: L5 Slope, 5: L5 Incidence, 6: Pelvic Incidence, 7: Pelvic Tilt, and 8: Sacral Slope).
4. Global sagittal alignment parameters: SVA (the horizontal offset from the posterosuperior corner of S1 to the vertebral body of C7), and T1 Slope (the angle between a horizontal line and the superior end plate of T1).
5. Slip percentage was assessed in those lumbar isthmic spondylolisthesis patients^32^. Figure 4 demonstrated the measurement of slip percentage.

**Figure 4:**
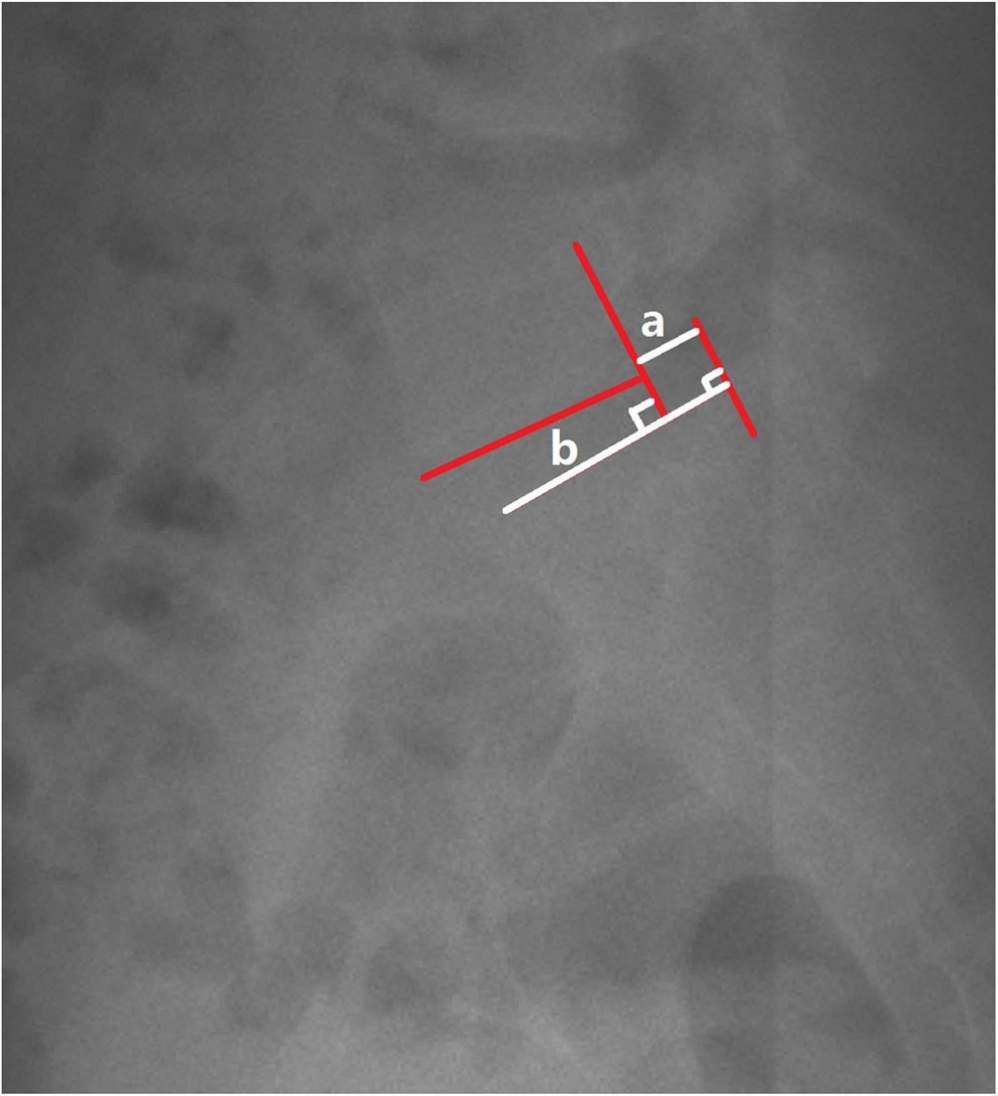
Demonstrated the measurement of slip percentage.

### Statistical analysis

Statistical analyses were performed using SPSS 19.0 statistics software (SPSS Inc., Chicago, IL). Descriptive statistics were listed in the form of mean ± SD (standard deviation). An independent-sample *t* test was employed to assess the difference between groups. Count data distribution was assessed by *Chi-square* test. Correlation analysis was performed to assess the associations between slip percentage and other parameters in the case group.

To identify the main risk factors of adolescent symptomatic isthmic spondylolisthesis, multiple logistic regression models were constructed using sagittal lumbosacral parameter variables and global sagittal alignment parameters that were of significance in univariate analysis. *P* < 0.05 was considered as the significant level.

In addition, there are gender differences in several aspects, such as growing speed, and skeletal structure. Subgroup analysis was also performed based on gender differences.

### Data availability statement

The data sets generated during the current study are available from the first author (Jian Zhao) on request.
